# Particulate Emissions: Health Effects and Labour Market Consequences

**DOI:** 10.1155/2012/130502

**Published:** 2012-07-29

**Authors:** Marie Kruse, Bjørn Sætterstrøm, Jakob Bønløkke, Henrik Brønnum-Hansen, Esben Meulengracht Flachs, Jan Sørensen

**Affiliations:** ^1^Centre for Applied Health Technology Assessment, University of Southern Denmark, J.B. Winsløwsvej 9B 1, 5000 Odense C, Denmark; ^2^The Danish Institute for Health Services Research, Dampfærgevej 27–29, 2100 Copenhagen, Denmark; ^3^Section of Environmental and Occupational Health, Institute of Public Health, Aarhus University, Bartholins Allé 2, 8000 Aarhus C, Denmark; ^4^Institute of Public Health, University of Copenhagen, Øster Farimagsgade 5, 1014 Copenhagen, Denmark; ^5^National Institute of Public Health, University of Southern Denmark, Øster Farimagsgade 5, 1014 Copenhagen, Denmark

## Abstract

The objective of this study was to analyse the productivity cost savings associated with mitigation of particulate emissions, as an input to a cost-benefit analysis. Reduced emissions of particulate matter (PM_2.5_) may reduce the incidence of diseases related to air pollution and potentially increase productivity as a result of better health. Based on data from epidemiological studies, we modelled the impact of air pollution on four different diseases: coronary heart disease, stroke, lung cancer, and chronic obstructive pulmonary disease. We identified individuals with these diseases and modelled changes in disease incidence as an expression of exposure. The labour market affiliation and development in wages over time for exposed individuals was compared to that of a reference group of individuals matched on a number of sociodemographic variables, comorbidity, and predicted smoking status. We identified a productivity cost of about 1.8 million EURO per 100,000 population aged 50–70 in the first year, following an increase in PM_2.5_ emissions. We have illustrated how the potential impact of air pollution may influence social production by application of a matched study design that renders a study population similar to that of a trial. The result suggests that there may be a productivity gain associated with mitigation efforts.

## 1. Introduction

Pollution has wide-ranging consequences, for health as well as in other sectors [1]. Following a Health Impact Assessment of particulate matter emissions [2], this study aims to quantify the labour market consequences of particulate emissions as part of a cost-benefit analysis of mitigation efforts. 

What are the costs and benefits of air pollution mitigation? The costs include the actual mitigation costs as well as the disutility (and costs) of those who are forced to reduce their emissions, while the benefits include the utility of a cleaner air as well as the benefits associated with lower levels of pollution. Among the benefits from reduced air pollution are the economic gains related to improved health. This study focuses on some of these gains, namely, the productivity gains from lower pollution-related morbidity and mortality. For practical reasons, the gains are quantified by means of assessing their opposite measure: the costs of increased pollution.

Air pollution impacts on health through increased incidence and mortality of a number of diseases [2]. In this analysis, we focus on the increase in incidence of coronary heart disease, stroke, lung cancer, and chronic obstructive pulmonary disease (COPD), all of which are associated with increased levels of fine particulate (PM_2.5_) emissions. Miller et al. [3] found that women who were exposed to an increase in the level of PM_2.5_ by 10 microgram per m^3^ had a relative risk (RR) of 1.21 (95% Cl: 1.04–1.42) of experiencing a coronary heart disease event and 1.35 (95% Cl: 1.08–1.68) of experiencing an episode of stroke. Similarly, Pope III et al. [4] found a RR of 1.14 (95% Cl: 1.04–1.23) for lung cancer in both genders when particulate emissions increased by 10 micrograms per m^3^. The RR for COPD related to particulate emissions was also 1.14 for a similar increase in both genders [5].

Based on findings on mortality related to particulate emissions [4, 6], we assumed that the RR for cardiovascular events following exposure to particulate emissions for men was lower than that for women. Tentatively, therefore, we assumed that the RR of cardiovascular disease in exposed men was 1.29 and the RR for coronary heart disease in men exposed to particulate emissions was 1.175. We had no reason to assume a similar gender difference for pulmonary diseases. Instead we applied similar RR estimates for the two genders relating to pulmonary diseases. 

When analysing health or social consequences of a risk factor such as air pollution in an epidemiological framework, it is necessary to identify exposed and nonexposed individuals. However, when examining the labour market consequences of air pollution, such an approach may not be feasible since all individuals in industrialised countries are exposed to some degree of air pollution. The health effects and the associated labour market consequences are often described in a dose-response relationship. Also, exposure may be related to transport habits and other parameters that are not easily measured at population level. Our approach to measuring exposure was therefore to express exposure to air pollution as the health effects of air pollution. More precisely, we developed a framework where the labour market consequences of diseases were assessed based on assumptions of the RR of disease incidence when exposed to air pollution. Basically, we considered the measurable health effects of air pollution as being an expression of overall exposure to air pollution.

We focused on incidence instead of prevalence because we wished to capture the effects of particulate emission-related disease on both morbidity and mortality. Merely focusing on prevalence would underestimate the mortality impact, because survivors make up a relatively larger share of the prevalent population, compared to their share of the incident population. This applies to any disease study, as simple mathematics dictate that since mortality is often higher in severe cases of a given disease, the prevalent population can be construed as relatively healthier than the incident population.

The aim of this paper was to quantify potential productivity gains due to improved health following a mitigation of air pollution through reduction of small particulate (PM_2.5_) emissions. Practically, we have approached this task by quantifying the productivity costs of health consequences of existing particulate emissions and assumed that this present cost can be saved when mitigation succeeds. In this study, we have applied known health effects of pollution on labour market behaviour. Health effects on labour market behaviour was either productivity costs due to death or early retirement, or relative wage losses, that is, workers with a disease or reduced health will receive a lesser wage increase.

The productivity costs due to mortality and premature departure from the labour market constitute the societal costs of disease, while the wage loss expresses a loss to the individual worker. A cost-benefit analysis will from a societal perspective include both cost components.

## 2. Materials and Methods

### 2.1. Selection of Study Population

We assessed the labour market consequences of the four diseases in a register-based analysis. We used disease as an expression of exposure and thus identified a patient group as “the exposed” and a control group as “the nonexposed.” The control group was identified such that they were as comparable to the patient group as possible. The difference in labour market affiliation and wage developments were considered as being attributable to disease and thus air pollution. In calculations of changes in costs, we assumed an increase in PM_2.5_ of 10 microgram/m^3^. 

Patients with at least one hospital admission caused by coronary heart disease (ICD10 diagnoses I20–25), stroke (ICD10: I60–69), lung cancer (ICD10: C33-34), or COPD (ICD10: J41–44) were identified in the Danish National Hospital Register. In addition, individuals that had died from these diseases but had not been hospitalised for the disease while alive were identified in the National Causes of Death Register. The identified patients were matched with a reference group of nonpatients, henceforth called the control group. Both groups were followed for a period of 10 years, with 1998 being the baseline year and 1999 the year of first hospital contact for coronary heart disease patients, lung cancer patients, and stroke patients. Due to hospitalisation occurring late in the disease history for COPD patients, we defined two groups of COPD patients, the first having their first hospitalisation in 1999, like the three other diseases, and the second having their first hospitalisation in 2006. The baseline year for both groups was 1998. We adopted a wash-out period of 5 years (1994–98) during which patients could not have had any health care contacts related to the analysed disease. The rationale of the wash-out period was to ensure that cases were in fact incident and had not been hospitalised for their disease previously. 

For each disease, we selected a control group amongst the entire adult Danish population, using individual, nearest neighbour matching by propensity scores [7, 8] comprising age in one-year intervals, comorbidities (a Charlson index [9] excluding the analysed disease), marital status, national origin, length of education, socioeconomic status, and area of residence. Matching was conducted in five-year age strata and gender. There were five controls per case. Controls with the analysed disease occurring before or after 1999 were excluded. For both patients and controls, we obtained data for annual, gross wages, and labour market affiliation from the national registers at Statistics Denmark. These register data were linked to health data via the social security number. Most labour market data reflects the labour market status of a person in November. Data allows, however, for adjustment for unemployment spells during the year. 

We used the variables displayed in Table 1 for analysis.

### 2.2. Labour Market Affiliation

For those patients that were in the labour market at baseline (1998), labour market affiliation was analysed using duration analysis (Cox regression). Individuals were considered to be in the labour market when they were employed, self-employed, or unemployed. If they deceased or received age pension, disability pension, or early retirement, they were considered outside the labour market. Other categories, such as students, were excluded. 

The duration model estimated the excess risk of leaving the labour market for patients compared to controls by modelling LM (labour market affiliation) as a function of Ds (disease) (see Table 1 for abbreviations of variables).

The resulting hazard ratio (HR) for the Ds parameter expresses the increased risk of leaving the labour market due to mortality or morbidity among patients, when compared to controls. Since confounding factors have been taken into account in the matching procedure, there is no need to apply adjustment in the model. Indeed, further adjustment would cause overadjustment for the covariates by including their effect twice. 

We tested whether the proportional hazards assumption was sustained, using the proportionality test option in PROC PHREG of SAS. 

### 2.3. Wage Consequences

Wage consequences were analysed using a difference-in-difference approach. For each patient, the wage development from 1998 to 2000 was recorded and compared to the wage development for the matched controls, using the same period. Only individuals with a labour market income in both years were included in this analysis. Hence, pensioners and others having only a nonlabour market income were excluded from this analysis of wage development. Also people leaving the labour market due to pension (or death) were excluded.

The crude wage development over the period 1998–2000 was compared between patients and controls. The wage loss related to disease was quantified as

(i) WLdisease=(Wagecontrol2000−Wagecontrol1998)−(Wagepatient2000−Wagepatient1998).

### 2.4. Productivity Costs of PM_2.5_

The increased risk of leaving the labour market due to disease, multiplied by the annual wage of a control person, constitutes the average productivity cost of leaving the labour market too soon due to disease. Similarly, the wage loss computed via ($\mathopen {}\oldleft (\text {ii}\oldright )\mathclose {}$) relates to the total wage loss due to disease. The productivity costs of particulate emission relating to both the labour market withdrawal and the wage loss are computed by multiplying the two with the elevated risk of disease due to particulate emission, that is, RR-1. The particulate emission related productivity costs per person are transformed to population-based figures using incidence of disease. For incidence, we have used data on first-ever hospital admissions and causes of death figures for individuals aged 50–70 years.

The productivity cost (PC_pollution_) of disease was estimated by multiplying the excess risk of disease incidence (RR-1) with the excess risk of leaving the labour market (HR-1) and the average wage of patients in 2000, being the first calendar year after the event:

(ii) $$\begin{array}{c} {\text{PC}}_{\text{pollution}}=\left(\text{RR}-\text{1}\right)*\left(\text{HR}-\text{1}\right)*{\text{Wage}}_{\text{patients}}2000. \end{array}$$

Similarly for wage differences,

(iii) $$\begin{array}{c} {\text{WL}}_{\text{pollution}}=\left(\text{RR}-\text{1}\right)*{\text{WL}}_{\text{disease}}. \end{array}$$

Equations ($\mathopen {}\oldleft (\text {ii}\oldright )\mathclose {}$) and ($\mathopen {}\oldleft (\text {iii}\oldright )\mathclose {}$) express labour market consequences per patient. In order to arrive at the socioeconomic gain of mitigation of particulate emissions, the labour market consequences per patient should be related to incidence as shown in the following:

(iv) number of saved cases=incidence before−incidence after=incidence before−(1RR)∗incidence before=(1-1RR)∗incidence before,

where RR is the relative risk of disease incidence when exposed to particulate emissions. Incidence figures are related to the age group 50–70, and results should therefore be interpreted as savings per 100,000 population aged 50–70. We computed the gain for one year and also accumulated the cost over a ten-year period. The results for the ten-year period were computed, considering that a smaller share of the population is in the labour market each year, due to labour market withdrawal in the preceding years. Also, future costs were discounted by 3 per cent per year in order to obtain the net present value. Cost figures are expressed in 2000-EURO's using an exchange rate of €1 = 7.5 DKK.

We used SAS v. 9.2 for all analyses. Individual data were stored at Statistics Denmark which ensured full compliance with all confidentiality laws and regulations.

## 3. Results

For all four diseases, there was a statistically significant higher risk of leaving the labour market for patients with the four diseases in comparison with nondiseased controls.  Table 2 displays the estimated hazard ratios for leaving the labour market early.

The impact of disease on labour market affiliation shown in Table 2 indicates that there is a productivity loss associated with these diseases. 

Table 3 displays wages at baseline (1998) and two years later (in 2000). Wage developments were used for calculation of difference-in-difference: did controls experience a better development in wages than patients? All wages were converted to 2000-fixed prices. From Table 3, it appears that there were only minor differences between patients and controls in terms of wage increases. The exception is lung cancer, where the disease apparently has a beneficial impact on wage development. It should be noted that, due to poor survival, very few lung cancer patients remain in the labour market after onset of disease, and those who do may not be typical.

When interpreting the results in Table 3, it should be noted that the figures only relate to the individuals who were still in the labour market. Those who left the labour market due to disease or death were excluded from these calculations. Hence, the population figures do not reflect the entire matched study population, but only those still in the labour market in 2000.

Table 4 summarises the productivity costs of an increase in particulate emissions at 10-microgram per m^3^ at patient and societal level. Hence, it combines the findings of Tables 2 and 3 on the productivity costs of disease to the impact of particulate emissions on these diseases. In Table 4, the productivity costs of disease are multiplied by the elevated risk of disease when exposed to increased particulate emissions. The resulting productivity costs from a 10-microgram increase in particulate emission are presented as the average per exposed person, per 100,000 population aged 50–70 years in the first year and per 100,000 population aged 50–70 years accumulated over ten years. The latter figure represents the net present value of one year's exposure. During the ten-year period, almost all patients have left the labour market. The results in the last two columns of Table 4 are computed according to ($\mathopen {}\oldleft (\text {iv}\oldright )\mathclose {}$) and hence represent a saving that can be harvested when mitigation efforts succeed.

In the first year after disease incidence, the productivity cost of a 10-microgram per m^3^ increase in particulate emission amounts to about 1.8 million EURO per 100,000 population in the age group 50–70. Assuming a linear effect, the accumulated impact of one year's incidence amounts to more than 9 million EURO over a ten-year period. 

## 4. Discussion 

We have found that pollution-related disease impacts on labour market affiliation, and therefore there is a societal gain from reducing air pollution. This finding points towards an important consequence of particulate emissions on labour market affiliation through health effects and hence indicates that a potential productivity gain may arise from successful mitigation of air pollution. To our knowledge, this topic has not been analysed by other authors.

We also looked at the wage development and compared patients suffering pollution-related disease and controls. We found no significant wage differences attributable to pollution-related disease. 

The analysis was designed as a comparison of a group of exposed individuals with a group of controls. The control group was selected using a propensity score approach, aiming at achieving a comparable control group with characteristics similar to the exposed group. By selecting a control group that was as similar to the patient group as possible, we aimed to analyse the counterfactual: what would have happened if they had not become ill. One challenge of this exercise is the selection of the characteristics that comprise the propensity score: that is, determining which characteristics of patients and controls are important in the analysis of labour market affiliation and wages. In the analysis, we have used a variety of available socioeconomic variables in the composition of the propensity score. 

Therefore, socioeconomic and demographic parameters that may impact on labour market behaviour should be captured by the matching procedure, at least to some extent. One of the disadvantages of the propensity score approach is that not all characteristics weigh the same in the score and more minor variations may not be captured. The variables that weigh the most in the propensity score are those contributing the most to explain disease occurrence. This weighing of variables is not possible when matching is conducted using individual variables, which was one of the reasons we did not adopt that approach. Another option of course would be regression analyses on the entire population including adjustment for relevant covariates. Such an approach could include application of instrumental variables [10], addressing potential unobservable individual effects. Though this may be part of explaining the relation between disease and labour market behaviour, we chose to focus on relative effects instead, in a trial-like setup. The similarity of wages before the incidence year indicates that matching was successful.

We have focused on a variety of characteristics in our matching procedure. These include age, gender, and length of education, but also socioeconomic status and comorbidity. Other variables may also be important in explaining the complex relation between pollution, health, and labour market behaviour. We suggest that the relation between labour market behaviour and health is as complex as reflected in our model, and perhaps even more so. While acknowledging this, we may tentatively conclude that a decrease in particulate emissions may be associated with reduced disease incidence and mortality which may have a positive impact on labour market affiliation and productivity.

The evidence of smoking as an important risk factor for disease dates several years back [11, 12]. Compared to smoking, particulate emissions only have marginal effect on disease development. This should be borne in mind, in particular when analysing diseases such as COPD and lung cancer, in which smoking is an important risk factor. If the studies that describe interactions between mortality and air pollution do not capture the effect of smoking as an effect modifier sufficiently, the impact of air pollution on health may be slightly overestimated. It is also possible that other risk factors interact with smoking and pollution exposure [13], causing some uncertainty in our findings. The actual magnitude of our results may thus be somewhat uncertain. Nevertheless, it remains clear that particulate emissions incur significant costs due to their health effects and that mitigation of emissions would be associated with a substantial saving to society. 

## 5. Conclusion

We have illustrated how the potential impact of air pollution may influence social production by application of a matched study design that renders a study population similar to that of a trial. We found that there is a significant productivity cost related to the health effects of pollution. These results suggest that there may be a productivity gain associated with mitigation of particulate emissions. 

Further economic research could incorporate our findings into a cost-benefit analysis of a given mitigation intervention. In addition, our findings could be supported by further epidemiological research into the association between air pollution and disease. 

## Figures and Tables

**Table 1:** Data structure.

Variable	Levels	Application in analysis
Ds: disease	1 if incident disease (first time event of CHD, stroke, or lung cancer in 1999 or COPD hospitalisation in 2006)	Main explanatory variable
Age	1-year age group	Included in propensity score
5-year age intervals	5-year age group	Used for stratification of matching procedure
Gender	0 if man; 1 if woman	Used for stratification of matching procedure
CMI: comorbidities	Diseases weigh from 1 to 6 (modified Charlson index)	Included in propensity score
Married: marital status	1 if married or cohabiting, 0 otherwise	Included in propensity score
Origin: national origin	0 if Danish; 1 if not Danish	Included in propensity score
Edu: length of education	1–8, where 8 is university degree	Included in propensity score
Socio: socioeconomic status at baseline (1998) People outside the labour market were excluded from this analysis	(1) Self-employed	Included in propensity score
	(2) White collar	
	(3) Blue collar	
	(4) Unemployed	
Residence	Regional code used as a proxy for urban/rural	Included in propensity score
LM: labour market affiliation	1 if employed or unemployed, 0 if retired or dead	Outcome variable
Wage	Continuous variable expressing the difference between wage in 2000 and wage in 1998	Outcome variable

**Table 2:** Risk of leaving the labour market earlier for patients compared to controls, by diagnosis.

Disease	Hazard ratio of leaving	95 percent confidence
	the labour market	interval for hazard ratio
Coronary heart disease	**1.129**	(1.124–1.133)
Stroke	**1.122**	(1.117–1.127)
Lung cancer	**1.355**	(1.342–1.37)
COPD	**1.14**	(1.134–1.147)

Hazard ratios that were statistically significant at *α* = 0.05 are indicated in bold.

**Table 3:** Average wage development 1998–2000, EURO-2000 price level.

Disease	Control group			Patient group			Difference in average wage development
	N 2000	Average wage 1998	Average wage 2000	N 2000	Average wage 1998	Average wage 2000
Coronary heart disease	17,219	28,930	29,913	2,898	28,197	29,100	80
Stroke	8,609	27,956	28,737	1,298	31,434	31,257	958
Lung cancer	2,571	26,706	27,154	110	25,746	27,816	−1,622
COPD	5,034	25,682	26,258	794	25,041	25,527	89

**Table 4:** Total productivity costs per 100,000 at risk—aged 50–70 (2000-€).

	Hazard ratio of leaving the labour market R^1^	Relative risk of disease^2^	Annual wage (2000 €)^3^	Annual average productivity cost €^4^	Annual average wage loss 2000 €^5^	Productivity cost first year €^6^	Accumulated productivity cost over ten years €^7^
Coronary heart disease	1.129	1.175	29,913	675	70	507,148	4,455,856
Stroke	1.122	1.29	28,737	1,017	842	877,430	7,709,195
Lung cancer	1.355	1.14	27,154	1,350	−1,046	278,009	2,442,619
COPD	1.14	1.14	26,258	515	77	140,843	1,237,461

Sources:

^1^Attributable risk of leaving the labour market: hazard ratios as presented in Table 2.

^2^Relative risk of disease when exposed to increased particulate emission, according to sources [3, 4].

^3^Annual wage in 2000 for controls, as in Table 3.

^4^The annual average productivity cost per patient per year 2000, computed according to (ii).

^5^The annual average wage loss in year 2000, according to (iii).

^6^First-year productivity cost per 100,000 population aged 50–70, computed according to (iv).

^7^Accumulated productivity cost per 100,000 population aged 50–70 over ten years. Computed as the findings of column 6, over a ten-year period, taking into account a declining labour market participation rate and a discount rate of 3 percent p.a.

## References

[1] Description of the CEEH integrated “Energy-environment-health-cost” modelling framework syste. http://ceeh.dk/CEEH_Reports/Report_1/index.html.

[2] CEEH scientific report no. 7a description of the CEEH health effect model. http://ceeh.dk/CEEH_Reports/Report_7a/index.html.

[3] Miller KA, Siscovick DS, Sheppard L (2007). Long-term exposure to air pollution and incidence of cardiovascular events in women. *New England Journal of Medicine*.

[4] Pope CA, Burnett RT, Thun MJ (2002). Lung cancer, cardiopulmonary mortality, and long-term exposure to fine particulate air pollution. *Journal of the American Medical Association*.

[5] Abbey DE, Lebowitz MD, Mills PK, Petersen FF, Beeson WL, Burchette RJ (1995). Long-term ambient concentrations of particulates and oxidants and development of chronic disease in a cohort of nonsmoking california residents. *Inhalation Toxicology*.

[6] Pope CA, Thun MJ, Namboodiri MM (1995). Particulate air pollution as a predictor of mortality in a prospective study of U.S. Adults. *American Journal of Respiratory and Critical Care Medicine*.

[7] Rubin DB (1997). Estimating causal effects from large data sets using propensity scores. *Annals of Internal Medicine*.

[8] Rosenbaum PR, Rubin DB (1983). The central role of the propensity score in observational studies for causal effects. *Biometrika*.

[9] Charlson ME, Pompei P, Ales KA, MacKenzie CR (1987). A new method of classifying prognostic comorbidity in longitudinal studies: development and validation. *Journal of Chronic Diseases*.

[10] Hausman JA, Taylor WE (1981). Panel data and unobservable individual effects. *Econometrica*.

[11] Jenkins CD, Rosenman RH, Zyzanski SJ (1968). Cigarette smoking. Its relationship to coronary heart disease and related risk factors in the Western Collaborative Group Study.. *Circulation*.

[12] Kannel WB, Castelli WP, McNamara PM (1968). Cigarette smoking and risk of coronary heart disease. Epidemiologic clues to pathogensis. The Framingham Study.. *National Cancer Institute monograph*.

[13] Villeneuve PJ, Goldberg MS, Burnett RT, van Donkelaar A, Chen H, Martin RV (2011). Associations between cigarette smoking, obesity, sociodemographic characteristics and remote-sensing-derived estimates of ambient PM_2.5_: results from a canadian population-based survey. *Occup Environ Med*.

